# Follicular Atresia in Buffalo: Cocaine- and Amphetamine-Regulated Transcript (CART) and the Underlying Mechanisms

**DOI:** 10.3390/ani14152138

**Published:** 2024-07-23

**Authors:** Chunyan Yang, Haiying Zheng, Ahmed Amin, Marwa S. Faheem, Anqin Duan, Lingyu Li, Peng Xiao, Mengqi Li, Jianghua Shang

**Affiliations:** 1 Guangxi Key Laboratory of Buffalo Genetics, Reproduction and Breeding, Guangxi Buffalo Research Institute, Chinese Academy of Agricultural Sciences, Nanning 530001, China; haiyingzheng@126.com (H.Z.); a.amin@agr.cu.edu.eg (A.A.); duanaq321@163.com (A.D.); lly01202020@163.com (L.L.); xiaopengger@163.com (P.X.); limengqi92@foxmail.com (M.L.); 2 Key Laboratory of Buffalo Genetics, Breeding and Reproduction Technology, Ministry of Agriculture and Rural Affairs, Nanning 530001, China; 3 Animal Production Department, Faculty of Agriculture, Cairo University, Giza 12613, Egypt; marwasf@agr.cu.edu.eg

**Keywords:** follicular atresia, buffalo, granulosa cells, apoptosis, cocaine- and amphetamine-regulated transcript (CART), AKT/GSK3β/β-catenin

## Abstract

**Simple Summary:**

In the present study, we aimed to explore the potential local regulatory role of the cocaine- and amphetamine-regulated transcript (CART) signaling pathway in granulosa cell (GC) apoptosis, which is a key mechanism promoting follicular atresia in several animal species, including buffalo. Our results showed how CART activity adversely influences buffalo GC viability by affecting estradiol production and enhancing apoptosis. The regulatory mechanism by which CART can affect GC apoptosis entails the modulation of the AKT/GSK3β/β-catenin pathway, a key intracellular signaling pathway essential for cell viability. In conclusion, this study provides valuable insights into the intricate mechanisms governing ovarian follicle development and granulosa cell function. These findings have implications for reproductive biology not only for buffalo but also for different species.

**Abstract:**

Atresia is a process in ovarian follicles that is regulated by hormone-induced apoptosis. During atresia, granulosa cell (GC) apoptosis is a key mechanism orchestrated through diverse signaling pathways. Cocaine- and amphetamine-regulated transcript (CART) signaling within ovarian GCs has been demonstrated to play a key role in the regulation of follicular atresia in cattle, pigs, and sheep. The present work aimed to investigate the potential local regulatory role of CART in GC apoptosis-induced follicular atresia in buffalo, focusing on the modulation of the AKT/GSK3β/β-catenin signaling pathways, which are the intracellular signaling pathways involved in cell viability. Our findings revealed increased expression of CARTPT and BAX and decreased levels of AKT, β-catenin, and CYP19A1 genes in atretic follicles compared to healthy follicles. Subsequently, CART treatment in the presence of FSH inhibited the FSH-induced increase in GC viability by reducing estradiol production and increasing apoptosis. This change was accompanied by an increase in the gene expression levels of both CARTPT and BAX. At the protein level, treatment with CART in the presence of FSH negatively affected the activity of AKT, β-catenin, and LEF1, while the activity of GSK3β was enhanced. In conclusion, our study shows how CART negatively influences buffalo GC viability, underlying the modulation of the AKT/GSK3β/β-catenin pathway and promoting apoptosis—a key factor in follicular atresia.

## 1. Introduction

During the reproductive life of mammals, a small percentage (less than 1%) of primary follicles are selected for ovulation, while the remaining 99% are prone to follicular atresia, in which they deteriorate and undergo apoptosis [1,2]. This phenomenon occurs in monovulatory species such as cattle and humans, which have the same ovarian follicular dynamics, where antral follicles grow in waves. Within each wave, a temporary increase in follicle-stimulating hormone (FSH) triggers the growth of recruited follicles. Among them, one follicle achieves dominance and continues to develop, while the others lose their steroidogenic ability, resulting in follicular atresia [3,4,5]. Follicular atresia is a hormonally directed process essential for maintaining normal ovarian function and involves the elimination of unnecessary structures and cells. Granulosa cells (GCs) functionally coordinate both the developmental potential of oocytes and steroid secretion either during the follicular phase or after ovulation within the follicular compartment [6]. Therefore, the apoptosis of ovarian GCs has been shown to be a major contributor to follicular atresia in various animal species, such as rats [7,8,9], mice [10], cattle [11,12], pigs [13], chickens [14,15], and humans [16], particularly at the antral stage of follicular development [17,18]. Since cell-specific apoptosis plays a vital role in cellular remodeling, differentiation, and development, understanding the underlying mechanisms and pathways that regulate GC apoptosis is highly important. This is because a deviant signal in one of these pathways might influence ovarian functions [19,20]. GC apoptosis is a complex biological process that is regulated by a delicate balance between pro- and antiapoptotic pathway activities. Various signaling pathways, such as protein kinase A (PKA), protein kinase B (PKB/AKT), extracellular signal-regulated kinase 1 and 2 (ERK1/2), p38 mitogen-activated protein kinase (p38-MAPK), epidermal growth factor (EGF), and bone morphogenetic proteins (BMPs), function as inhibitors of GC apoptosis. These pathways modulate the activity of more than 100 target genes [21]. Conversely, suppressing PKB/AKT leads to the nuclear localization of the transcription factor forkhead box O (FoxO), which induces GC apoptosis and follicular atresia by activating genes such as Bcl-2-like protein 11 (Bim), tumor necrosis factor-related apoptosis-inducing ligand (TRAIL), and Fas ligand (FasL) [22,23,24]. Consequently, considerable progress has been made in understanding the regulatory mechanisms of GC apoptosis during follicular atresia in different animal species [25]. Nevertheless, there is a shortage of information and reports underlying the mechanisms and pathways that govern GC apoptosis in buffalo species, particularly during follicular development and atresia. One of the intrinsic regulatory factors that has not been previously investigated in buffalo GC apoptosis is cocaine- and amphetamine-regulated transcript (CART). CART is a neuropeptide released by hypothalamic neurons that participate in controlling various physiological functions, such as feeding behavior, energy metabolism, hormone release, stress response, anxiety, depression, bone resorption, and reproduction [26,27]. Cocaine- and amphetamine-regulated transcript mRNA and peptide were detected in bovine oocytes and ovarian cells, specifically in the cumulus and granulosa cell layers of antral follicles. However, preantral follicles do not exhibit CART expression, implying a potential involvement of CART in the atresia mechanism of antral follicles [28]. CART might promote follicular atresia either by inhibiting follicular growth and development or by inducing GC apoptosis. Supporting this notion, concentrations of CART in the follicular fluid decline following the selection of a dominant follicle, and the levels of CARTPT mRNA are lower in healthy follicles than in atretic follicles prior to and during the early stages of follicle dominance [29]. Furthermore, CART exerts a potent inhibitory effect on GC stimulation by FSH and IGF1 under in vitro conditions and significantly reduces the in vivo production of follicular estradiol (E2), negatively influencing follicular development and inducing atresia [30]. This is particularly important since bovine GC estradiol production in response to FSH is MAPK- and AKT-dependent [31]. Sen’s work revealed that CART inhibits FSH-stimulated Ca2+ influx, cAMP accumulation, and MAPK and AKT signaling pathway activity, ultimately reducing CYP19A1 mRNA expression and E2 production [32]. This converging evidence might support a novel local role for CART in regulating GC apoptosis, which is a key factor in the induction of follicular atresia in various animal species, including cattle [29,30], sheep [33], and pigs [34]. Although the potential role of CART in bovine GC apoptosis during follicular atresia has been extensively investigated, whether CART plays a similar role in buffalo has yet to be determined. Considering the similarity of the CART gene between buffalo and bovine GCs, which exceeds 99%, we hypothesized that CART may act as a potential local regulator in the process of buffalo GC apoptosis through further investigation of the association between CART activity and GC apoptosis in relation to follicular atresia in buffalo.

## 2. Materials and Methods

### 2.1. Experimental Design

To address our hypothesis, we first investigated the association between CART gene (CARTPT) expression and follicular atresia. For that purpose, the expression levels of the CART gene were determined in both healthy and atretic follicles. In addition, the expression levels of the target genes involved in apoptosis (BAX and BCL2), cell survival and proliferation (AKT and β-catenin), and the CYP19A1 gene, which is known to be responsible for converting androgen to estrogen, were evaluated. Second, we examined the effect of FSH, which is known as the primary survival factor for antral follicles, alongside CART supplements on GC estradiol production. Third, to address whether CART can influence GC steroidogenesis and regulate cell apoptosis in buffalo, we established an in vitro model in which GCs were cultured under four different treatment conditions: either alone (0×FSH) or supplemented with CART (0×FSH+CART), and two additional groups of GCs cultured in medium containing FSH, either in the absence (1×FSH) or presence (1×FSH+CART) of CART. In all the studied groups, the main function of the GCs, E2 production, was assessed. Gene expression analysis of CARTPT, BAX, BCL2, AKT, β-catenin, and CYP19A1 and protein expression of AKT, p-AKT, GSK3β, p-GSK3β, β-catenin, and LEF1 (lymphoid-enhancer-binding factor 1; coupling with β-catenin to regulate cell survival [35]) were also performed. In addition, apoptosis was evaluated in all the experimental groups.

### 2.2. Materials, Supplies, and Chemicals

All plasticware used in this study was purchased from Nunc (Wiesbaden, Germany), while all chemicals and media were purchased from Sigma-Aldrich Chemical (St. Louis, MO, USA); with the exception of antibodies, fetal bovine serum (FBS) and DMEM were purchased from Gibco BRL (Paisley, Scotland, UK). All studies and procedures were reviewed and approved by the Animal Ethics Committee of the Guangxi Water Buffalo Research Institute (AECGXBRI).

### 2.3. Classification of Follicles as Healthy or Atretic and Sample Collection

Buffalo ovaries, 20 to 30 ovaries, were collected from adult animals at a local abattoir and transported to the laboratory (within 3 h) in sterile 0.9% NaCl solution at 25–30 °C. Follicles with a diameter greater than 8 mm were used to collect follicular fluid for E2 and progesterone (P4) assays. Using previously defined criteria for assessing follicle health, a practical classification system was established based on the relative levels of E2 and P4 found in the follicular fluid [36,37]. Follicles with an E2/P4 ratio < 1 were classified as atretic follicles, while follicles with an E2/P4 ratio > 5 were considered healthy follicles. Next, GCs were pooled from 20 to 30 follicles per group and preserved in Trizol for mRNA extraction, facilitating the comparison of gene expression levels between healthy and atretic follicles.

### 2.4. Assessment of Estradiol and Progesterone Levels in Follicles

The concentrations of E2 and P4 in follicular fluids and of E2 in GC culture medium were evaluated using commercially available enzyme-linked immunosorbent assay (ELISA) kits supplied by Fankewei (Shanghai, China) according to the manufacturer’s instructions. The assay sensitivity was 0.5 pmol/L, the inter- and intra-assay coefficients of variation (CVs) for E2 were 8.2 and 7.9%, respectively, and the inter- and intra-assay CVs for P4 were 9.4 and 5.6%, respectively. Six replicates were examined for each tested condition, and the results are presented as the concentrations of the steroids (pmol/L).

### 2.5. RNA Isolation and Complementary DNA (cDNA) Synthesis

Total RNA from buffalo GCs was extracted using Trizol^®^ reagent (TaKaRa, Dalian, China) and dissolved in RNase-free water. DNAse digestion was performed to remove any genomic DNA from the extracted samples. A constant amount of RNA (100 ng) was then directly reverse-transcribed into 20 μL of first-strand cDNA by using the PrimeScript RT Reagent Kit with gDNA Eraser (Perfect Real Time, TAKARA BIO Inc., Shiga, Japan) according to the instructions provided by the manufacturer. Finally, the resulting cDNA was stored at −20 °C until further use.

### 2.6. Cloning and Homology Assessment of Buffalo CART Partial cDNA

A pair of specific primers was designed based on conserved regions found in the nucleotide sequence from the National Center for Biotechnology Information GenBank Nucleotide database for reverse transcription–polymerase chain reaction analysis of CARTPT mRNA expression in the buffalo ovary (Table 1). Specimens of buffalo ovarian stroma were obtained from three different animals. A partial CART cDNA fragment was amplified from the buffalo samples by using a TaKaRa Ex Taq kit (TaKaRa, Dalian, China). The thermal cycler program consisted of 35 cycles at 98 °C for 10 s, 57 °C for 30 s, and 72 °C for 1 min. The amplified cDNA was subsequently purified by using a TIAN Gen Mini Purification Kit (TIANGEN Biotech; Beijing Co., Ltd., Beijing, China) and cloned and inserted into the pMD19-T vector (TaKaRa). Next, the plasmids containing inserts were sequenced, and alignment of the nucleotide sequences was performed with the NCBI BLAST program “http://www.ncbi.nlm.nih.gov/BLAST (accessed on 21 January 2022)”.

### 2.7. Relative Quantification of Gene Expression

The relative expression levels of CART mRNA and related genes in both healthy and atretic follicles and in buffalo GCs cultured under different in vitro culture conditions were measured via quantitative real-time polymerase chain reaction (qRT–PCR). Reactions were performed in a total volume of 20 μL and contained an equal amount of cDNA (100 ng), 10 mM each of the forward and reverse primers (the primer sequences of genes used in this experiment were designed via the Primer-Blast online tool, http://www.ncbi.nlm.nih.gov/tools/primer-blast (accessed on 21 January 2022), and listed in Table 1, and 10 μL of 2× SYBR Green Master Mix (SYBR^®^ Premix Ex Taq™ II, TAKARA, Japan). All reactions for all the genes of interest were performed in triplicate and run on a Light Cycler 480 system (Roche Diagnostics, Atlanta, GA, USA) under the following conditions: 95 °C for 30 s, followed by 40 cycles of 95 °C for 5 s and 58 °C for 30 s. The relative expression of all the target genes was normalized to that of the endogenous control reference genes β-actin and GAPDH and analyzed with the 2^−ΔΔCT^ method. Before performing the quantitative RT-PCR for the selected genes, the stability of both endogenous genes (β-actin and GAPDH) was confirmed by investigating the consistency of their expressions among the experimental samples.

### 2.8. GC Collection, Culture, and Treatment

After ovaries were collected, they were washed with a new saline solution supplemented with 1000 U/mL penicillin and 1000 μg/mL streptomycin. Follicular fluid containing GCs was aspirated from buffalo follicles with a diameter of 8 mm by using an 18-gauge needle attached to a 10 mL injection syringe, and the GCs were collected by differential centrifugation as described previously [38]. GCs obtained from at least 10 ovaries were utilized in each replication to ensure an adequate cell number for different experiments. The collected GCs were then pooled in a 15 mL tube containing DMEM medium supplemented with ITS + 3 (2 µL/mL), IGF-1 (1 × 10^−6^ M), androstenedione (1 × 10^−7^ M), insulin (10 ng/mL), BSA (0.1%), nonessential amino acids (1.1 mM), and antibiotics (100 IU/mL penicillin and 0.1 mg/mL streptomycin). The cells were washed twice with culture medium and resuspended in 2 mL of medium. Then, the cell number and viability were estimated by using trypan blue exclusion. The determination of GC purity was confirmed based on the presence of follicle stimulating hormone receptor (FSHR), a marker specific to GCs, and the absence of CYP17A1, a marker specific to theca cells.

To explore the effect of either FSH and/or CART on GC function, GCs at a concentration of 2 × 10^5^ viable cells/well were seeded in 24-well plates and cultured in DMEM medium supplemented with 10% FBS, penicillin (100 U/mL), and streptomycin (100 μg/mL) at 38.5 °C in a humidified atmosphere of 5% CO_2_ in air. The cells were grown until they reached 40–50% confluence. The cells were washed twice and incubated in a fresh medium supplemented with different FSH concentrations (0, 0.25, 0.5, 1, 2, and 4 ng/mL) (Sigma-Aldrich, Waltham, MA, USA) for 24 h, after which the E2 concentration in the culture medium was measured. The optimum concentration of FSH was subsequently used to culture GCs with different concentrations (0, 12.5, 25, and 50 μM) of CART (13240; Sino Biological, Beijing, China) for 24 h, after which the E2 concentration in the collected culture medium was evaluated. The resultant optimum concentrations of both FSH and CART were used in the established culture system of GCs in all further investigations. To investigate the effect of CART on GC steroidogenesis and apoptosis in the presence or absence of FSH, a GC culture model was established. For that, cells (2 × 10^5^ viable cells/well) were cultured in 24-well plates under a humidified atmosphere of 5% CO_2_ in air at 38.5 °C. The medium was changed every two days. On the sixth day of culture, the GCs were cultured in medium alone (0×FSH), medium supplemented with CART (25 μM; 0×FSH+CART), or medium supplemented with FSH (1 ng/mL) in the presence (1×FSH+CART) or absence of CART (1×FSH) for 24 h. The media were collected and stored at −80 °C for subsequent measurement of E2 levels. The cells were washed two times with Dulbecco’s PBS. Afterward, the cultured GCs were subjected to various morphological assessments or harvested using trypsin EDTA and preserved at −80 °C for subsequent analysis. All the experiments were replicated at least three times.

### 2.9. Cell Apoptosis Assay

Cell apoptosis was assessed using the One Step TUNEL Apoptosis Assay Kit (Green Fluorescence, product code: C1086; Beyotime Biotechnology, Shanghai, China) following the manufacturer’s instructions. In brief, the cells from the treated groups were gently washed with PBS, followed by fixation in 4% paraformaldehyde for 15 min at room temperature. Subsequently, the cells were washed again and exposed to a permeabilization solution (freshly prepared with 0.1% Triton X-100 in 0.1% sodium citrate) for 2 min on ice. After a brief rinse with PBS, the cells were incubated with the TUNEL reaction mixture in a humidified chamber at 37 °C for 1 h. After a second brief PBS rinse, the cells were counterstained with DAPI for 8 min to visualize the nuclei. Stained GCs were observed and evaluated with a fluorescence microscope (Nikon Corporation, Tokyo, Japan) to determine the proportion of TUNEL-positive cells.

### 2.10. Immunofluorescence Analysis of AKT and β-Catenin Proteins

Approximately 50,000 viable cells were seeded in an 8-well chamber slide (Corning Incorporated, Kennebunk, ME, USA) and cultured in DMEM medium supplemented with 10% FBS, penicillin (100 U/mL), and streptomycin (100 μg/mL) at 38.5 °C in a humidified atmosphere of 5% CO_2_ until 40–50% confluency. After treatments, cells from each treatment group were subjected to the immunofluorescence analysis procedure described in a previous study [39], with some modifications. In brief, the GCs were washed three times with warm PBS and then fixed in a solution of 4% paraformaldehyde in PBS for 1 h at room temperature. The cells were then washed another three times with PBS, permeabilized with 0.2% Triton X-100 (Sigma-Aldrich) in PBS for 20 min at RT, and blocked by incubation at RT for 1 h with PBS supplemented with 1% BSA. Afterward, the GCs were incubated overnight at 4 °C with a specific primary antibody against AKT (1:500 dilution, 10176-2-AP; Proteintech, San Diego, CA, USA) and β-catenin (1:100 dilution, ab32572; Abcam, Cambridge, UK). The next day, the GCs were washed three times with 0.05% Tween 20 (P9416; Sigma-Aldrich) in PBS and incubated in the dark with fluorescein-conjugated goat anti-rabbit (1:500 dilution, ab150077; Abcam) secondary antibody at 37 °C for 1 h. After washing twice with PBS, the nuclei were stained with Hoechst 33342 (5 mg/mL) for 5 min. Finally, the cells were washed twice again to remove excess Hoechst. Fluorescence was detected using the green and blue fluorescence of a Nikon microscope (Nikon Corporation, Tokyo, Japan).

### 2.11. Western Blot Analysis of the Tested Proteins

Western blotting (WB) was performed as described previously [40]. In brief, GCs from each group were harvested, washed twice with cold PBS, and lysed in RIPA buffer containing PMSF (R0010; Solarbio, Beijing, China) at 4 °C for 30 min. This was followed by centrifugation at 12,000 rpm for 5 min at 4 °C. The lysates were subsequently diluted with 6× protein loading buffer (DL101-02; TransGen, Beijing, China) and heated to 100 °C for 5 min. After cooling on ice, the samples were loaded on a 12% gradient polyacrylamide gel (P0012AC; Beyotime, Shanghai, China), transferred to a polyvinylidene difluoride (PVDF) membrane (ISEQ00011; Millipore, Shanghai, China), and blocked in 8% (*w*/*v*) Difco Skim Milk in Tris-buffered saline containing 0.1% (*v*/*v*) Tween-20 (TBST) for 2 h. As the molecular weights of the tested proteins, including β-Catenin (85 kDa), GSK3β (46 kDa), phosphorylated GSK3β (p-GSK3β) (46 kDa), AKT (56 kDa), P-AKT (60 kDa), LEF1 (44 kDa), and β-actin (43 kDa), were different from each other, each half was probed with a separate primary antibody. The membrane was incubated overnight at 4 °C with monoclonal anti-β-catenin (Abcam, ab32572), anti-GSK3β (Abcam, ab227208), p-GSK3β (CST, 9336), anti-AKT (Proteintech, 10176-2-AP), P-AKT (CST, 9271), anti-LEF1 (Abcam, ab22884), and a control protein, β-actin (Abcam, ab8226), diluted 1:1000 in blocking buffer. After washing three times with TBST for 15 min each, the membranes were incubated for 1 h at 37 °C with anti-rabbit IgG (Proteintech, SA00001-2) for GSK3β, p-GSK3β, AKT, P-AKT, LEF1, and β-catenin, while goat anti-mouse IgG (Abcam, ab205719) was used for β-actin as a secondary antibody at a dilution of 1:1000 in blocking buffer. The membranes were then washed three times in TBST (Tris Buffered Saline + Tween 20) again and developed using ECL Plus (Beyotime, P0018). Finally, the blot bands were detected with a multifunction imager (Syngene, Cambridge, UK). The intensities of individual bands were normalized to the expression of β-actin. WB was performed three times.

### 2.12. Statistical Analyses

A minimum of three biological replicates were used in each experiment. The normality of the distribution of the data was examined. All the data were analyzed using the SPSS 11.0 software program (SPSS, Chicago, IL, USA). The statistical differences between the treatment groups were calculated as the mean expression levels of the genes and proteins tested, either in granulosa cells or in healthy and atretic follicles of buffalo, and hormone concentrations, either in follicular fluids or in culture media, were analyzed via one-way ANOVA. All the data are expressed as the means ± SDs of three biological replicates. In addition, multiple stat corrections to the *p*-value were performed using the Bonferroni adjustment approach, and probability values less than 0.05 (*p* < 0.05), 0.025 (*p* < 0.025), and 0.0125 (*p* < 0.0125) were considered to indicate statistical significance.

## 3. Results

### 3.1. Analysis of CART Nucleotide Sequence Homology between Buffalo and Other Species

**Figure 1:**
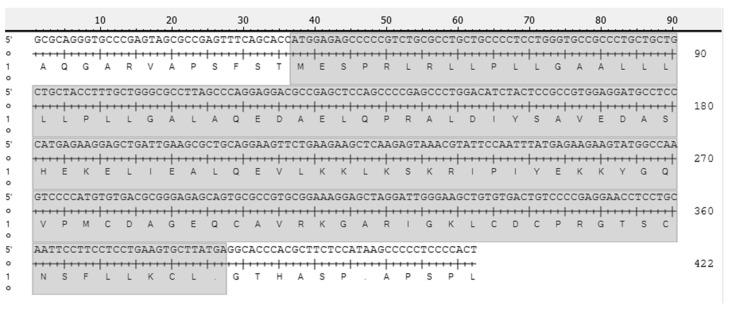
Cloning and sequencing of the water buffalo CART gene cDNA and predicted protein encoding.

To identify the similarity in CART gene sequence between buffalo and other species, a total length of 351 bp was amplified from the cDNA of the CDS region of ovarian buffalo. Cloning and sequencing of the water buffalo CART gene cDNA were subsequently performed. The results of the sequencing and prediction of the encoded proteins are presented in Figure 1.

The obtained buffalo sequences were compared and analyzed against the gene sequences of domestic cattle, American bison, wild yak, sheep, Bactrian camel and dromedary camel, macaque monkeys, horses, donkeys, humans, and wild boar in GenBank using NCBI Sequence Similarity Analysis “https://blast.ncbi.nlm.nih.gov/Blast.cgi (accessed on 25 July 2022)”. The reference sequences and similarity analysis results are shown in Table 2. The obtained buffalo CART gene sequence showed 100% similarity to domestic cattle and American bison, 98% similarity to wild yak and sheep, 91% similarity to bactrian camel and dromedary camel, 90% similarity to macaque monkeys, 85% similarity to horses and donkeys, 81% similarity to humans, and 78% similarity to wild boar.

### 3.2. Expression Patterns of CART and Related Genes in Relation to Follicle Health Status

**Figure 2:**
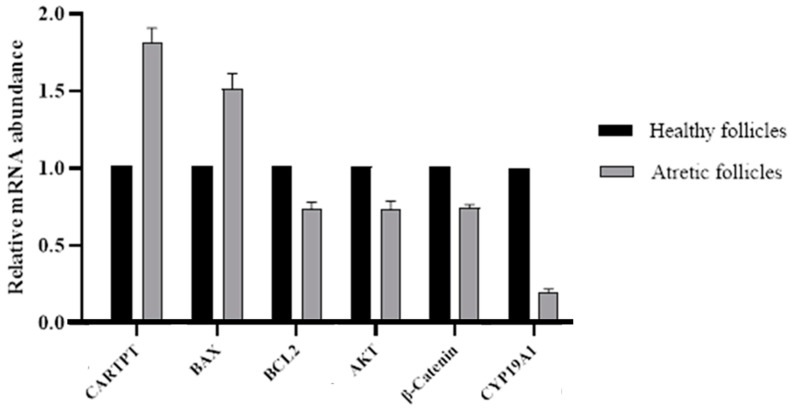
Relative abundance (expressed as fold change) of CARTPT, BAX, BCL2, AKT, β-Catenin, and CYP19A1 genes in buffalo GCs derived from atretic (*n* = 28 per group) compared to healthy (*n* = 30 per group) follicles. Expression data of target genes were normalized to the expression levels of β-actin and GAPDH and analyzed using the comparative CT (2^ΔΔCT^) method. Data are shown as means ± SD, *p* < 0.025 as compared to the control healthy follicles group, and are representative of at least three biological replicates.

To determine whether the health status of buffalo follicles is associated with the activity of CART in GCs, we first investigated the mRNA expression levels of CARTPT in GCs isolated from both healthy and atretic follicles. Analysis of the qRT–PCR data revealed that, compared with those from the GCs of healthy follicles, those from atretic follicles exhibited significantly (*p* < 0.025) greater CARTPT mRNA expression (Figure 2).

Second, we examined the expression levels of genes involved in apoptotic pathways, including BAX and Bcl2, as well as genes associated with cell survival and proliferation, namely, AKT and β-catenin. Our results showed that the mRNA abundance of BAX was notably (*p* < 0.025) greater in GCs derived from atretic follicles than in those derived from healthy follicles. Conversely, BCL2 exhibited an opposite trend to BAX. Moreover, the mRNA levels of AKT and β-catenin were greater in healthy follicles than in atretic follicles. Finally, our investigation focused on assessing the mRNA expression of the aromatase enzyme encoding the CYP19A1 gene, which exhibited a higher level of activity in GCs found within healthy compared to atretic follicles.

### 3.3. Effects of Different Doses of CART and/or FSH on Estradiol Production in Cultured Buffalo GCs In Vitro

**Figure 3:**
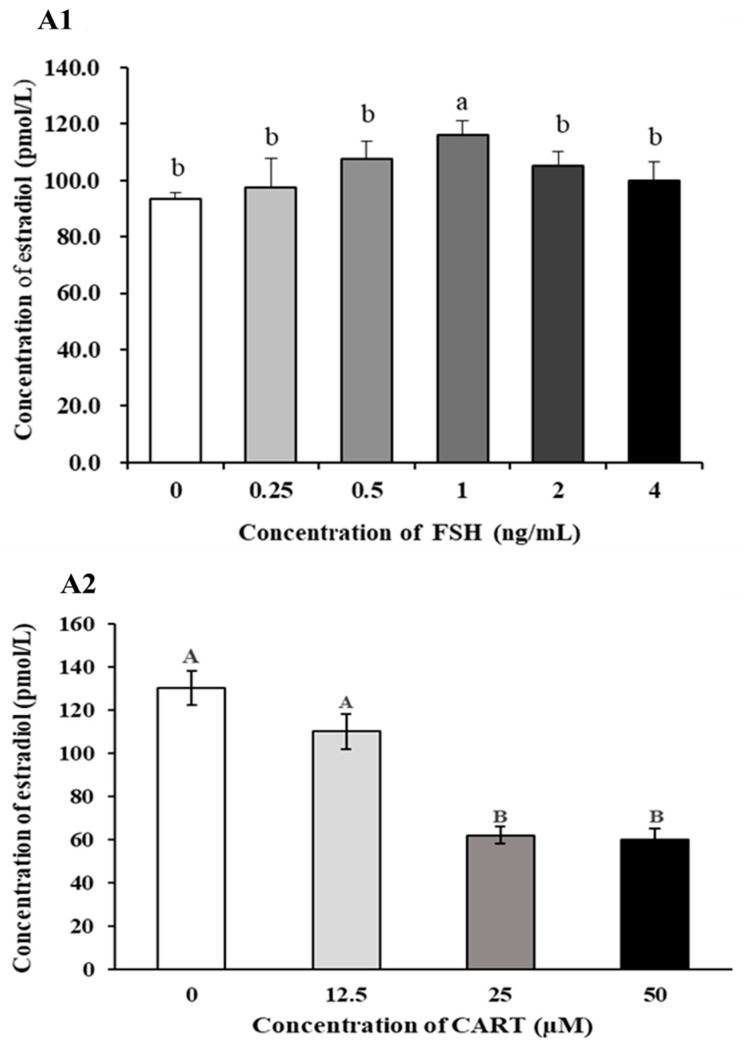
Responsiveness of buffalo GCs to different doses of FSH (**A1**) and CART (**A2**) in the presence of FSH (1 ng/mL) with respect to estradiol levels in culture medium. Superscript small and capital letters indicate statistically significant differences between treatments (*p* < 0.05).

To verify the effect of CART and/or FSH treatment on E2 production in buffalo GCs, the optimal stimulatory dose of FSH was determined for the cultured GCs. To test this hypothesis, an experiment was conducted to assess the responsiveness of GCs to various FSH concentrations (0, 0.25, 0.5, 1, 2, and 4 ng/mL). The maximum increase in E2 accumulation occurred at a concentration of 1 ng/mL FSH (Figure 3(A1)). Consequently, this specific dosage was chosen for all subsequent experiments conducted within the in vitro culture system of GCs. It shows that the production of estradiol in the granulosa cells of buffalo ovarian follicles requires the induction of FSH. The dose-response effect of CART on estradiol production was then investigated in the presence of the optimal concentration of FSH (1 ng/mL). Our findings indicated a decrease in the concentration of E2 in the GC culture medium as the concentration of CART increased (0, 12.5, 25, and 50 μM). In addition, the production of E2 was significantly suppressed when GCs were pretreated with 25 μM CART (*p* < 0.05) (Figure 3(A2)).

### 3.4. Effect of CART on Estradiol Production in the Presence or Absence of FSH

**Figure 4:**
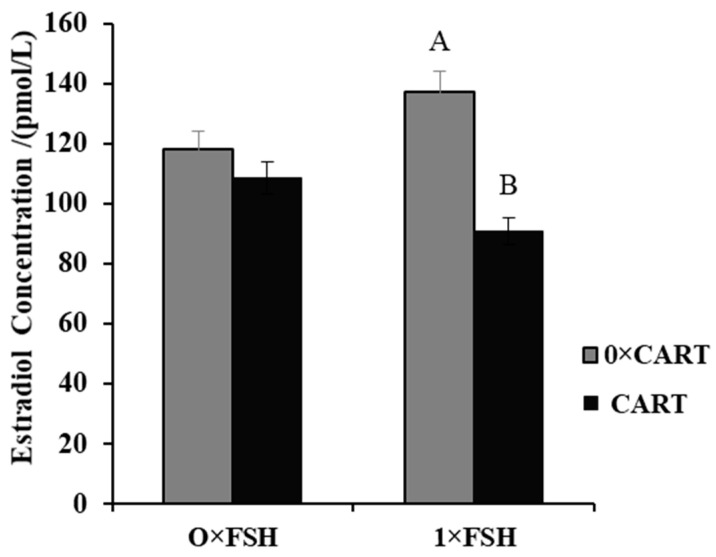
Effect of CART on buffalo GC estradiol production in the presence and absence of FSH. Superscript capital letters indicate statistically significant differences between the experimental groups (*p* < 0.025).

To investigate the impact of CART on buffalo GC steroidogenesis, the E2 concentration in buffalo GC culture medium supplemented with or without CART (25 µM) in the presence or absence of FSH (1 ng/mL) was assessed. As a result, in the absence of FSH, supplementation with CART had no effect on E2 production (118.23 ± 23.64 vs. 108.58 ± 27.41 pmol/L). However, supplementation with 25 µM CART significantly (*p* < 0.025) decreased estradiol production (137.05 ± 15.72 vs. 90.93 ± 18.19 pmol/L) in the presence of FSH (Figure 4).

### 3.5. Treatment with CART Increases Apoptosis in Buffalo GCs

**Figure 5:**
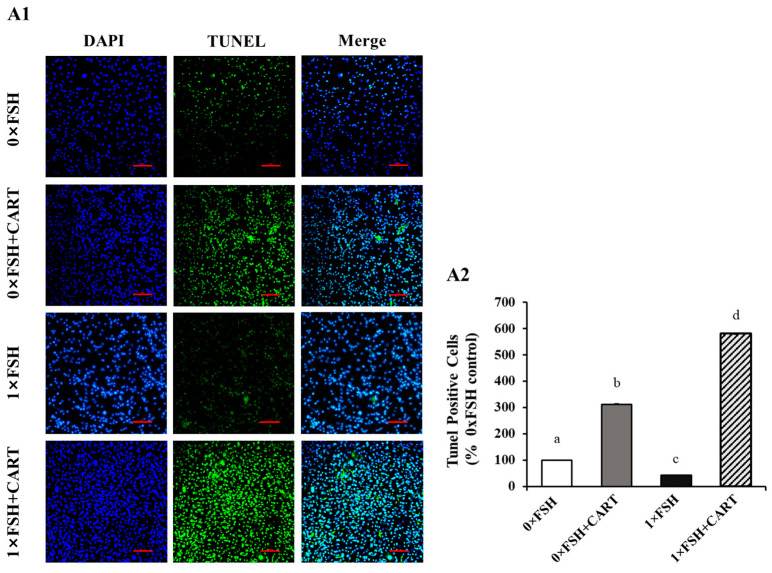
Analysis of apoptosis in the buffalo GCs. (**A1**) Representative images of TUNEL staining are shown. Blue staining (DAPI) indicates the nuclei of cells, and green fluorescent staining indicates the TUNEL-positive cells. (**A2**) The percentage of TUNEL-positive nuclei of GCs in the treated groups compared to the 0×FSH control group is presented. Superscript small letters indicate statistically significant differences among the experimental groups (*p* < 0.0125).

TUNEL staining was used to detect apoptosis in the GCs. CART supplementation, either alone or in combination with FSH, had a significant impact on apoptosis in buffalo GCs compared to that in the CART null groups, either in the 0×FSH or 1×FSH groups, as depicted in Figure 5. In addition, the 1×FSH+CART-treated group of GCs exhibited significantly elevated apoptosis levels compared to those of the other studied groups, while the FSH group (1×FSH) exhibited the lowest level of apoptosis (*p* < 0.0125).

### 3.6. GC Treatment with CART Alters the Expression Patterns of Genes Involved in Cell Survival, GC Steroidogenesis, and Apoptosis

To determine whether the adverse effect of CART on E2 production extends to the underlying functional genes involved in cell apoptosis, survival, and GC steroidogenesis function, the expression patterns of the CARTPT, BAX, BCL2, AKT, and CYP19A1 genes were monitored in the cultured GCs from the same experimental groups.

**Figure 6:**
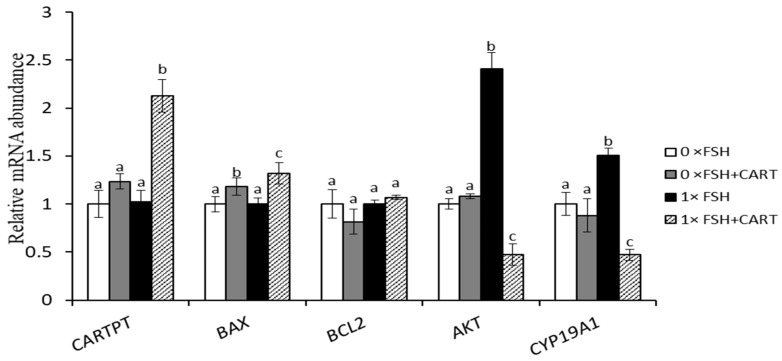
Relative mRNA abundance of CARTPT, BAX, BCL2, AKT, and CYP19A1 in GCs cultured in vitro with or without CART in the presence or absence of FSH. Expression data of target genes were normalized to the expression levels of β-actin and GAPDH and analyzed using the comparative CT (2^ΔΔCT^) method. Data are presented as the mean ± SD of three biological replicates. Different superscript letters (a, b, and c) denote a significant difference between the experimental groups (*p* < 0.0125).

As shown in Figure 6, CART treatment significantly (*p* < 0.0125) increased the expression of the CARTPT and BAX genes in the presence of FSH (1×FSH+CART group). However, there were no significant differences in the BCL2 expression level either in the presence or absence of FSH in response to CART treatment.

In addition, CART treatment inhibited the expression of both AKT, the key gene in the AKT/GSK3β/β-catenin signaling pathway, and CYP19A1 in the presence of FSH. In contrast, the group treated with FSH alone (1×FSH group) exhibited significant increases in the mRNA levels of AKT and CYP19A1 compared to those in the other groups (*p* < 0.0125).

### 3.7. Differential Modulation of the AKT/GSK3β/β-Catenin Signaling Pathway by CART in the Presence or Absence of FSH

**Figure 7:**
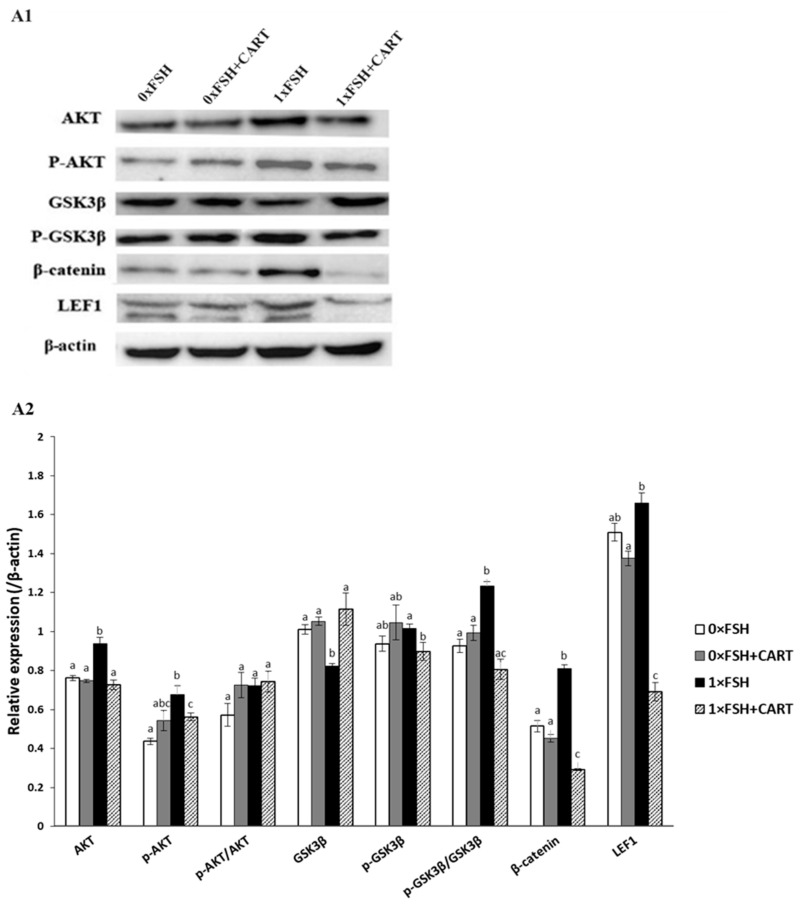
Effect of granulosa cell culture medium supplementation with CART on the protein expression levels of AKT, p-AKT, GSK3β, p-GSK3β, β-Catenin, and LEF1. Protein expression was analyzed by western blot (**A1**). The densitometric analysis of the immunoblots was performed (**A2**). Superscript small letters indicate significant differences; values with the same letters are not significantly different, and values with different letters are significantly different (*p* < 0.0125).

To ascertain the underlying mechanism of CART for regulating GC apoptosis, WB and immunofluorescence were conducted to confirm the expression levels of AKT/GSK3β/β-catenin signaling pathway-related proteins. Figure 7 shows the differences in the western blot patterns of changes in total and phosphorylated AKT, total and phosphorylated GSK3β, β-catenin, and LEF1 in the GC experimental groups. Our data indicated that the protein level of AKT increased significantly in the FSH-treated group (1×FSH), while no significant differences were observed among the other studied groups (*p* < 0.0125). Similarly, compared with the other treatments, FSH alone significantly increased the protein expression of pAKT, while the addition of CART to FSH dramatically decreased the positive effect of FSH alone on the activity of both AKT and pAKT. On the other hand, treatment with CART did not alter the expression of the pAKT protein in the absence of FSH. Furthermore, CART treatment had no effect on the pAKT/AKT ratio, either in the presence or absence of FSH (*p* < 0.0125).

WB analysis of GSK3β and p-GSK3β protein expression in buffalo GCs revealed notable differences in the modulation patterns across treatments. The GSK3β protein levels were consistently greater in the 0×FSH, 0×FSH+CART, and 1×FSH+CART groups than in the 1×FSH group, and these differences were significant (*p* < 0.0125). Moreover, the levels of p-GSK3β in buffalo GCs exhibited somewhat different profiles. When CART was supplemented without FSH (0×FSH+CART), there was a slight increase in p-GSK3β levels, but this increase was not significantly greater than that in the 0×FSH control group. However, when FSH was combined with CART (1×FSH+CART), a notable decrease in p-GSK3β levels was observed compared to that in the 1×FSH group. The ratio of p-GSK3β to total GSK3β was significantly greater in the FSH group (1×FSH) than in the other groups (*p* < 0.0125). However, the addition of CART to the 1×FSH group dramatically attenuated the impact of FSH alone, resulting in a decreased p-GSK3β/GSK3β ratio in the 1×FSH+CART group. Conversely, CART alone did not significantly affect the GCs cultured in the absence of FSH (*p* < 0.0125). Analysis of β-catenin protein expression revealed that, compared with those in the 0×FSH control group, the expression of β-catenin in the GCs treated with 1×FSH was significantly greater. Treatment with CART in the presence of FSH (1×FSH+CART) resulted in a marked decrease in β-catenin expression compared to that in the other groups (*p* < 0.0125).

Furthermore, there was a slight increase in LEF1 protein expression in the 1×FSH group compared to the 0×FSH group, although the difference was not statistically significant (*p* < 0.0125). However, the inclusion of CART in combination with FSH resulted in a modest reduction in LEF1 expression compared to that in the untreated control group (0×FSH); this reduction reached a noticeably significant level in the absence of FSH (*p* < 0.0125).

### 3.8. CART Regulation of the FSH-Stimulated AKT/GSK3β/β-Catenin Signaling Pathway in Buffalo GCs at the Protein Level

**Figure 8:**
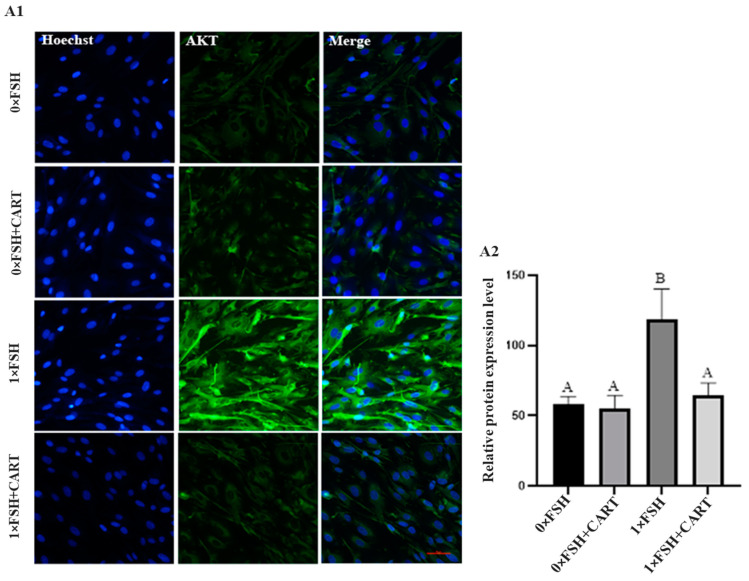
Representative images of buffalo granulosa cells immunolabeled for AKT (green) and Hoechst-stained cells’ nuclei (blue) (**A1**). For negative control, only secondary antibodies were used. Red bars represent 100 µm. AKT protein expression was quantified by immunofluorescence staining of AKT (**A2**). Data are presented as the mean ± SD of three biological replicates. Different superscript letters (A and B) denote a significant difference between the tested groups (*p* < 0.0125).

Further investigations into the potential role of CART in regulating the AKT/GSK3β/β-catenin signaling pathway were conducted by examining the protein expression of AKT and β-catenin in four distinct groups of buffalo GCs using immunofluorescence analysis. The results revealed significant differences in AKT protein expression among the treatment groups (*p* < 0.0125), as illustrated in Figure 8. In line with our previous WB results, the FSH treatment group (1×FSH) presented significantly greater levels of AKT protein expression than did the 0×FSH and 0×FSH+CART groups. Additionally, the addition of CART in combination with FSH (1×FSH+CART) resulted in a significant decrease in AKT protein expression compared to that in the FSH-only group (1×FSH) (*p* < 0.0125).

**Figure 9:**
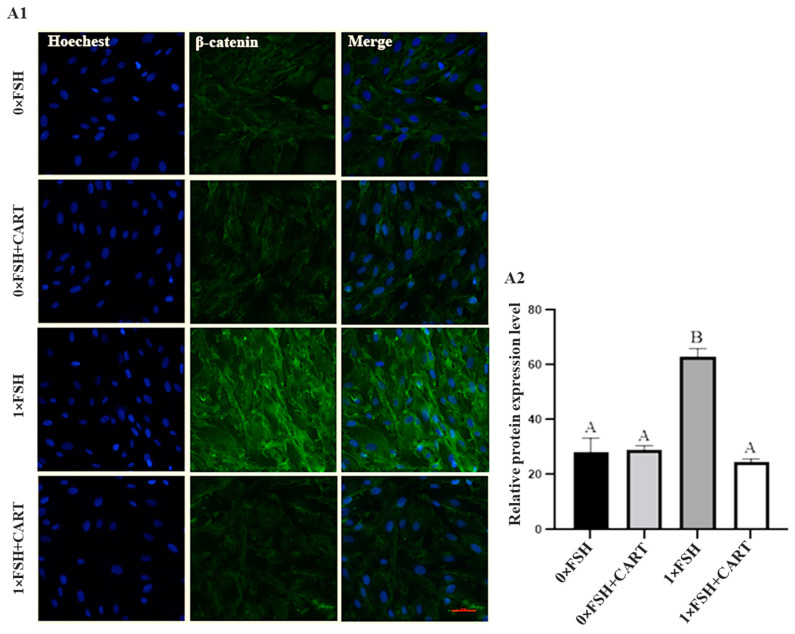
Representative images of buffalo granulosa cells immunolabeled for β-catenin (green) and Hoechst-stained cells’ nuclei (blue) (**A1**). For negative control, only secondary antibodies were used. Red bars represent 100 µm. β-catenin protein expression was quantified by immunofluorescence staining of AKT (**A2**). Data are presented as the mean ± SD of three biological replicates. Different superscript letters (A and B) denote a significant difference between the tested groups (*p* < 0.0125).

The IHC results for β-catenin showed that cells treated with FSH (1×FSH) exhibited a pronounced increase in β-catenin protein expression compared to that in the other groups, as illustrated in Figure 9, demonstrating a highly significant effect (*p* < 0.0125). This finding indicates that FSH plays a substantial role in the activation of β-catenin in buffalo granulosa cells. Interestingly, when CART was presented alongside FSH (1×FSH+CART) in the GC culture medium, β-catenin expression significantly decreased compared to that in the other groups. Conversely, the addition of CART to the GC culture medium in the absence of FSH (0×FSH+CART group) did not significantly change β-catenin expression compared to that in the untreated group (0×FSH). These findings demonstrated that CART may counter the promotive effect of FSH on AKT/GSK3β/β-catenin pathway activity by dysregulating the expression of both the AKT and β-catenin proteins.

## 4. Discussion

Most of the ovarian follicle reservoirs in female mammals do not reach full maturation upon transitioning from the resting stage to the growth phase. Since follicular development depends on sustained production of estradiol by ovulatory follicles, more than 99% of ovarian follicles have been found to lose their capacity to produce estradiol; instead, they undergo atresia. The regulation of follicle growth, development, and even atresia involves a complex interplay of various endocrine and intraovarian factors [6]. While there is a comprehensive understanding of the endocrine and molecular regulatory mechanisms that govern follicular growth and development, our understanding of the inhibitory factors and their underlying mechanisms that adversely affect this process, ultimately leading to atresia, is limited, especially for some adaptive species, such as buffalo.

The potential regulatory mechanism of CART during mammalian follicular development and atresia was first investigated in 2004 [41]. To date, evidence supports an important role for CART as an inhibitory factor during follicular development in several animal species, such as cattle [29], pigs [34], and sheep [33]. However, there is a lack of evidence regarding the extent of its contribution to ovarian follicle evolution in certain species, including buffalo, as well as the responsiveness of GCs in such species to CART. This study, for the first time, demonstrated an association between CART activity and follicular health status in buffalo. Here, we have conducted important investigations to determine the mRNA expression levels of CART in buffalo GCs derived from both healthy and atretic follicles. The present results revealed that GCs from atretic follicles expressed significantly greater levels of the anorectic neuropeptide CARTPT mRNA compared to GCs from healthy follicles. In contrast, atretic follicles presented a significantly lower level of CYP19A1 mRNA, the key regulator of estradiol production. Previous studies have indicated that, during follicular development, FSH acts upon GCs to stimulate the expression of CYP19A1, which in turn facilitates the conversion of androgens into estradiol [42]. Estradiol is a steroid hormone that regulates major developmental events during ovarian follicle growth and maturation. Elevated levels of estradiol found in the follicular fluid of healthy follicles actively contribute to the selection of the dominant preovulatory follicle [43]. Conversely, follicular atresia has been associated with a decrease in the production of estradiol [44]. In the present study, our results indicated a notable reduction in the concentration of estradiol secreted by buffalo GCs cultured in vitro under a constant level of FSH as the concentration of CART added to the culture medium increased. In addition, treatment of GCs with CART alone, without FSH, reduced the estradiol concentration without significant effect. These findings solidify the notion that CART exerts inhibitory effects on estradiol synthesis in buffalo GCs, particularly during follicular atresia. Similarly, previous findings in porcine have shown a decrease in estradiol production when CART levels were increased in follicular GCs cultured under the same FSH concentration, suggesting an inhibitory effect of CART on porcine GC steroidogenesis [34]. In bovine species, prior studies have established a negative association between the expression of CARTPT and follicle health status by examining follicles at random stages of the follicular wave from abattoir sources [41]. Furthermore, evidence of the strong inhibitory effects of CART treatment on various components of the FSH signal transduction pathway has been provided, ultimately resulting in a decrease in estradiol production by GCs after in vitro culture [31,32]. Additionally, a recent study revealed the same role for CART within ovine follicular tissues, which exerts an inhibitory effect on sheep follicular growth and development [33]. On the basis of our findings, we found a high degree of similarity between the CART gene in buffalo and that in other species, as shown in Table 2. These findings collectively provide convincing evidence to support the notion that CART likely plays a similar negative regulatory role during ovarian follicle development in buffalo, reflecting its role in line with that of other species.

The potential negative effect of CART may be due to its suppressive effect on GC viability and proliferation and/or its ability to promote cell apoptosis [34]. AKT is known to regulate cell viability and apoptosis in various cell types [45]. Activation of AKT prevents apoptosis by inhibiting GSK3β, which has important implications for the survival and proliferation of human embryonic stem cells [46]. Moreover, prior studies offer compelling evidence that AKT is involved in follicular development and atresia. In this regard, inhibition of the PI3K/AKT pathway has been shown to trigger apoptosis in rat GCs [47]. In addition, when rat GCs were exposed to an AKT inhibitor in combination with FSH, the rate of apoptosis and cell death was greater than that in cells treated with FSH alone. These findings suggest that AKT inhibition promotes apoptosis in GCs, ultimately contributing to follicular atresia [48].

In contrast, AKT activity has been documented to be essential for FSH-induced expression of CYP19A1 and promotion of granulosa cell proliferation [49]. During the present study, we observed that as CARTPT mRNA levels increased, the expression of AKT and CYP19A1 decreased in buffalo atretic follicles, and the expression of the apoptotic gene BAX increased significantly compared to that in healthy follicles. Thus, we hypothesized that CART may serve as a potential local regulator in the process of buffalo GC apoptosis during follicular atresia by exerting its influence on the inhibition of estradiol production through negative regulation of AKT/GSK3β/β-catenin signaling pathway activity. Our established in vitro model of buffalo GCs demonstrated that treatment of GCs with CART in the presence of FSH led to a significant increase in the apoptosis rate and diminished the stimulatory effect of FSH on GC viability in terms of estradiol production. These findings were consistent with previous studies conducted in pigs [34], cattle [29], and sheep [33], which demonstrated the same ability for CART to inhibit cell viability and proliferation and induce apoptosis in ovarian follicle GCs during follicular atresia. However, these studies did not provide a clear explanation for the mechanism by which CART regulates GC apoptosis.

In the present study, the increase in the apoptosis rate observed in the presence of CART was concomitant with the upregulation of apoptosis-associated gene (BAX) expression and the downregulation of genes associated with cell viability and steroidogenesis, namely, AKT and CYP19A1. At the protein level, WB analysis revealed that the addition of FSH to the culture medium significantly increased AKT and pAKT levels, while the addition of CART diminished these effects and led to significant decreases in AKT and pAKT expression levels. GSK3β, a protein-serine/threonine kinase that is expressed in various tissues and cells, is a key downstream protein in the AKT/GSK3β/β-catenin pathway. Accumulating evidence suggests that GSK3β plays a crucial role in regulating cell survival, differentiation, and apoptosis in various eukaryotic species [50]. We speculated that CART may regulate the AKT/GSK3β/β-catenin pathway by controlling GSK3β activity, which has been previously identified as a physiological target of AKT [51]. GSK3β activity is regulated through phosphorylation, which acts as an inhibitory mechanism. In our current investigation, we found that FSH treatment, which activated AKT at both the mRNA and protein levels, resulted in increased phosphorylation of GSK3β, as evidenced by a greater ratio of p-GSK3β to total GSK3β, leading to GSK3β deactivation and a decreased apoptosis rate. These results were consistent with previous findings that activation of AKT stimulates phosphorylation of GSK3β, which was found to be involved in protection against stress-induced apoptosis in myoblasts via the PI3K/AKT/GSK3β signaling pathway in Rat-1 and PC12 cells [52]. However, in the present study, when CART was introduced alongside FSH, it significantly attenuated the impact of FSH, resulting in reduced phosphorylation of GSK3β, as indicated by a decreased p-GSK3β/GSK3β ratio and, concurrently, an increase in GSK3β activity. This change in GSK3β activity corresponded with an elevated rate of apoptosis in buffalo GCs, revealing the underlying mechanism through which CART might act. In addition, we found that activation of GSK3β decreased both β-catenin and LEF-1 protein expression in parallel. The inactivation of GSK-3β is known to stabilize β-catenin and its subsequent accumulation in the nucleus. This accumulation then triggers the expression of the known β-catenin-associated transcription factor, LEF-1 [53,54]. On the other hand, GSK3β can phosphorylate β-catenin, targeting it to the proteasome for degradation and preventing its translocation to the nucleus, as well as blocking its subsequent effects [55]. Collectively, these results suggest that AKT might affect the expression of both β-catenin and LEF-1 through targeting GSK3β in buffalo GCs.

## 5. Conclusions

In conclusion, this study provides complete intrinsic insight into the inhibitory effects that CART exerts on buffalo GC viability, particularly in terms of estradiol production, as well as promoting GC apoptosis, which is the key driver of follicular atresia incidence. This regulatory impact is achieved, at least in part, through the modulation of AKT/GSK3β/β-catenin cell viability pathway activity. These findings contribute to a better understanding of the regulatory mechanisms underlying the developmental capacity of ovarian follicles concomitant with GC function and may have implications for reproductive biology in buffalo and other species.

## Figures and Tables

**Table 1:** Real-time PCR primer data.

Gene Symbol	Primer Sequence (5′-3′)	Product Size (bp)	Accession No.
BAX	F:GTCTGAAGCGCATCGGAGAT R:GATGGTCCTGATCAACTCGGG	224	XM_025269476.1
BCL2	F:AGCGGGAGTTCAGTGTGACT R:AATCGGATGCACTCGTTAGG	118	XM_025273634.1
AKT	F:AAGAGGCAGGAGGAGGAGAC R:CCCAGCAGCTTCAGGTACTC	140	NM_001290841.1
CYP19A1	F:GCTTTTGGAAGTGCTGAACC R:ATCCAGTGAGCAGCAGGACT	98	XM_025295054.1
CARTPT	F:CTCTGAGCTCTTGCCCATCT R:TGCGCTCCCACCTTTTATAG	104	XM_006078190.2
β-Catenin	F: GATACCCAGCGTCGTACATC R: TCCTTGTCCTGAGCAAGTTC	242	XM_025272907.3
β-actin	F: GTCACCAACTGGGACGACAT R: GGTCTCGAACATGATCTGGGT	153	XM_025274489.3
GAPDH	F: CCTGCCAAGTATGATGAGA R: AGGTAGAAGAGTGAGTGT	130	XM_006065800.4

**Table 2:** Similarity analysis of the water buffalo CARTPT gene coding region with 11 species.

Species	E-Value/Random Matching Possibility	Similarity to CART Buffalo Gene (%)	Reference Sequence
Domestic Cattle (*Bos taurus*)	0	100	AY603972.1
American Bison (*Bison bison*)	7.00 × 10^−107^	100	XM_010853569.1
Wild Yak (*Bos mutus*)	0	98	CP027088.1
Sheep (*Ovis aries*)	4.00 × 10^−99^	98	XM_015101190.1
Bactrian Camel (*Camelus bactrianus*)	7.00 × 10^−74^	91	XM_010949313.1
Dromedary Camel (*Camelus dromedarius*)	1.00 × 10^−73^	91	XM_010974976.1
Macaque Monkey (*Macaca mulatta*)	2.00 × 10^−57^	90	NM_001265877.1
Horse (*Equus caballus*)	2.00 × 10^−54^	85	XM_023618226.1
Donkey (*Equus asinus*)	1.00 × 10^−53^	85	XM_014851944.1
Human (*Homo sapiens*)	3.00 × 10^−180^	81	NG_015988.1
Wild Boar (*Sus scrofa*)	0	78	EF581838.1

## Data Availability

The datasets used and/or analyzed during the current study are available from the corresponding author on reasonable request.
